# Supplementary material for: Oat (Avena sativa L.) Sprouts Restore Skin Barrier Function by Modulating the Expression of the Epidermal Differentiation Complex in Models of Skin Irritation
Source: Int J Mol Sci. 2023 Dec 8;24(24):17274. doi: 10.3390/ijms242417274 (PMC10743458; doi:10.3390/ijms242417274)
Supplement: Supplementary file 1 [file ijms-24-17274-s001.zip › ijms-2738812-supplementary.pdf]

Table S1. Scoring system for gross appearance of ACD mouse model.

Character of the lesions (COL)	Score
No lesion present	0
Excoriations only or with small punctuate crust, mild dryness	1
Multiple or coalescing punctuate crusts, moderate dryness	2
Erosion/ulceration, severe dryness or lichenification	3

Area % of the lesions (AOL)	Score
No lesion present	0
< 30%	1
30~60%	2
60~100%	3

*Score for ACD mouse model = {(COL+AOL)/6}×100

Table S2. Antibodies used in flow-cytometry.

Antibody	Company	Catalog number	Concentration(mg/mL)
CD4	BioLegend	100438	0.2
CD8a	BioLegend	100734	0.2
CD45	BioLegend	103116	0.2
CD19	BioLegend	152410	0.2
CD3	BioLegend	100216	0.5

Table S3. Primer sequences used in qPCR analysis.

Gene	Species		Sequence (5'-3')	Product size (bp)	Accession number
IL-1b	mouse	F	GCAACTGTTCCTGAACTCAAC	89	XM_006498795
		R	ATCTTTTGGGGTCCGTCAACT		
IL-5	mouse	F	TGGGGGTACTGTGGAAATGC	172	NM_010558
		R	TCAGCCTCAGCCTTCCATTG		
IL-6	mouse	F	CTTCCATCCAGTTGCCTTCTTG	142	NM_001314054
		R	AATTAAGCCTCCGACTTGTGAA		
IL-10	mouse	F	CAGAGAAGCATGGCCCAGAA	129	XM_036162094
		R	GCTCCACTGCCTTGCTCTTA		
FLG	mouse	F	CTGGGAGGCAAGCTACAACA	116	XM_017319842
		R	CATGGGATGACTGGGGTTCC		
IVL	mouse	F	CACCTGACCCAGAGTTGTCC	97	XM_006501047
		R	TCAGGTGGCTTCTGATGCTG		
LOR	mouse	F	CACCAGAAAAAGCAGCCCAC	152	NM_008508
		R	CAGCTAGAGCCTCCTCCAGA		
GAPDH	mouse	F	ACCAGAAGACTGTGGATGG	170	NM_001411843
		R	CACATTGGGGGTAGGAACAC		
IL-6	human	F	AGTGAGGAACAAGCCAGAGC	111	XM_054358146
		R	ATTTGTGGTTGGGTCAGGGG		
IL-8	human	F	GGTGCAGTTTTGCCAAGGAG	183	AK311874
		R	TTCCTTGGGGTCCAGACAGA		
IFNG	human	F	TGGCTTTTCAGCTCTGCATC	117	NM_000619
		R	CCGCTACATCTGAATGACCTG		
TNF	human	F	AACCTCCTCTCTGCCATCAA	125	NM_000594
		R	CTGAGTCGGTCACCCTTCTC		
GAPDH	human	F	GTCTCCTCTGACTTCAACAGCG	131	NM_001357943
		R	ACCACCCTGTTGCTGTAGCCAA		

Table S4. Antibodies used in immunoblotting.

Antibody	Company	Catalog number	Molecular weight (kDa)
IL-1β	FineTest	FNab04209	39
IL-5	Santacruz	sc-398334	23, 43
IL-6	GeneTex	GTX110527	30
IL-10	Bioss	BS-0698R	18
profilaggrin	Santa Cruz	sc-66192	180
filaggrin	Santa Cruz	sc-66192	41
loricrin	Abcam	ab85679	59
involucrin	Thermo Fisher	MA5-11803	150
STAT3	Cell signalling	30835	78
p-STAT3	Cell signalling	9145	78
ERK1/2	Cell signalling	4695	42, 44
p-ERK1/2	Cell signalling	4370	42, 44
SAPK/JNK	Cell signalling	9252	46, 54
p-SAPK/JNK	Cell signalling	4668	46, 54
p-p38	Cell signalling	4511	43
beta-actin	Cell signalling	4970s	45
Rabbit IgG	Cell signalling	7074S	-
Mouse IgG	Cell signalling	7076S	-
